# Long-Term Course of Humoral and Cellular Immune Responses in Outpatients After SARS-CoV-2 Infection

**DOI:** 10.3389/fpubh.2021.732787

**Published:** 2021-09-27

**Authors:** Julia Schiffner, Insa Backhaus, Jens Rimmele, Sören Schulz, Till Möhlenkamp, Julia Maria Klemens, Dorinja Zapf, Werner Solbach, Alexander Mischnik

**Affiliations:** ^1^Center for Infection and Inflammation Research, University of Luebeck, Luebeck, Germany; ^2^German Center for Infection Research (DZIF), Standort Hamburg-Borstel-Luebeck-Riems, Luebeck, Germany; ^3^Health Protection Authority, Luebeck, Germany; ^4^Medical Faculty, Centre for Health and Society, University Hospital, Institute of Medical Sociology, Heinrich-Heine-University, Düsseldorf, Germany; ^5^Institute for Experimental Immunology, Affiliated to EUROIMMUN Medizinische Labordiagnostika AG, Luebeck, Germany

**Keywords:** COVID-19, SARS-CoV-2, immunoglobulin, IgG, seroprevalence, interferon-gamma release assay, IGRA

## Abstract

Characterization of the naturally acquired B and T cell immune responses to severe acute respiratory syndrome coronavirus 2 (SARS-CoV-2) is important for the development of public health and vaccination strategies to manage the burden of COVID-19 disease. We conducted a prospective, cross-sectional analysis in COVID-19 recovered patients at various time points over a 10-month period in order to investigate how circulating antibody levels and interferon-gamma (IFN-γ) release by peripheral blood cells change over time following natural infection. From March 2020 till January 2021, we enrolled 412 adults mostly with mild or moderate disease course. At each study visit, subjects donated peripheral blood for testing of anti-SARS-CoV-2 IgG antibodies and IFN-γ release after SARS-CoV-2 S-protein stimulation. Anti-SARS-CoV-2 immunoglobulin G (IgG) antibodies were positive in 316 of 412 (76.7%) and borderline in 31 of 412 (7.5%) patients. Our confirmation assay for the presence of neutralizing antibodies was positive in 215 of 412 (52.2%) and borderline in 88 of 412 (21.4%) patients. Likewise, in 274 of 412 (66.5%) positive IFN-γ release and IgG antibodies were detected. With respect to time after infection, both IgG antibody levels and IFN-γ concentrations decreased by about half within 300 days. Statistically, production of IgG and IFN-γ were closely associated, but on an individual basis, we observed patients with high-antibody titres but low IFN-γ levels and vice versa. Our data suggest that immunological reaction is acquired in most individuals after natural infection with SARS-CoV-2 and is sustained in the majority of patients for at least 10 months after infection after a mild or moderate disease course. Since, so far, no robust marker for protection against COVID-19 exists, we recommend utilizing both, IgG and IFN-γ release for an individual assessment of the immunity status.

## Introduction

Infection with severe acute respiratory syndrome coronavirus 2 (SARS-CoV-2) leads to various symptoms, including cough, fever, cold, and loss of smell and taste. The course of the disease varies in symptoms and severity, from asymptomatic infections to severe pneumonia with lung failure and death. Manifestation indices are estimated to be 55–85% (1). About 48% of patients are women, and 52% are men. In Germany, 2.6% of all persons with confirmed SARS-CoV-2 infections died in connection with a COVID-19 illness. The main risk factors for death are age and comorbidities such as diabetes or obesity. The diagnosis is based on clinical grounds and proven by virus detection through rt-PCR in respiratory samples.

SARS-CoV-2 infects human cells by using the viral spike (S) protein, which binds to the angiotensin-converting enzyme-2 (ACE-2) receptor on host cells (2). The S-Protein is the immunodominant epitope that induces B and T cell responses upon natural infection (3, 4) and vaccination (5). Antibodies target the virus and can block infection and, thus, are an essential correlate of protection (6, 7). Likewise, T-lymphocytes contribute to protection through specific interactions with B cells and cytokine responses (8).

In this study, we analyzed the long-term course of the immune response with respect to serum IgG antibodies and the capacity of peripheral blood cells to produce interferon-gamma (IFN-γ) upon viral S-protein specific stimulation. On the basis of the previous experience of our group (9) with the low-diagnostic significance of IgA and IgM antibodies in the long-term course of infection, we deliberately investigated only IgG antibodies.

## Materials and Methods

### Study Area

**Figure 1:**
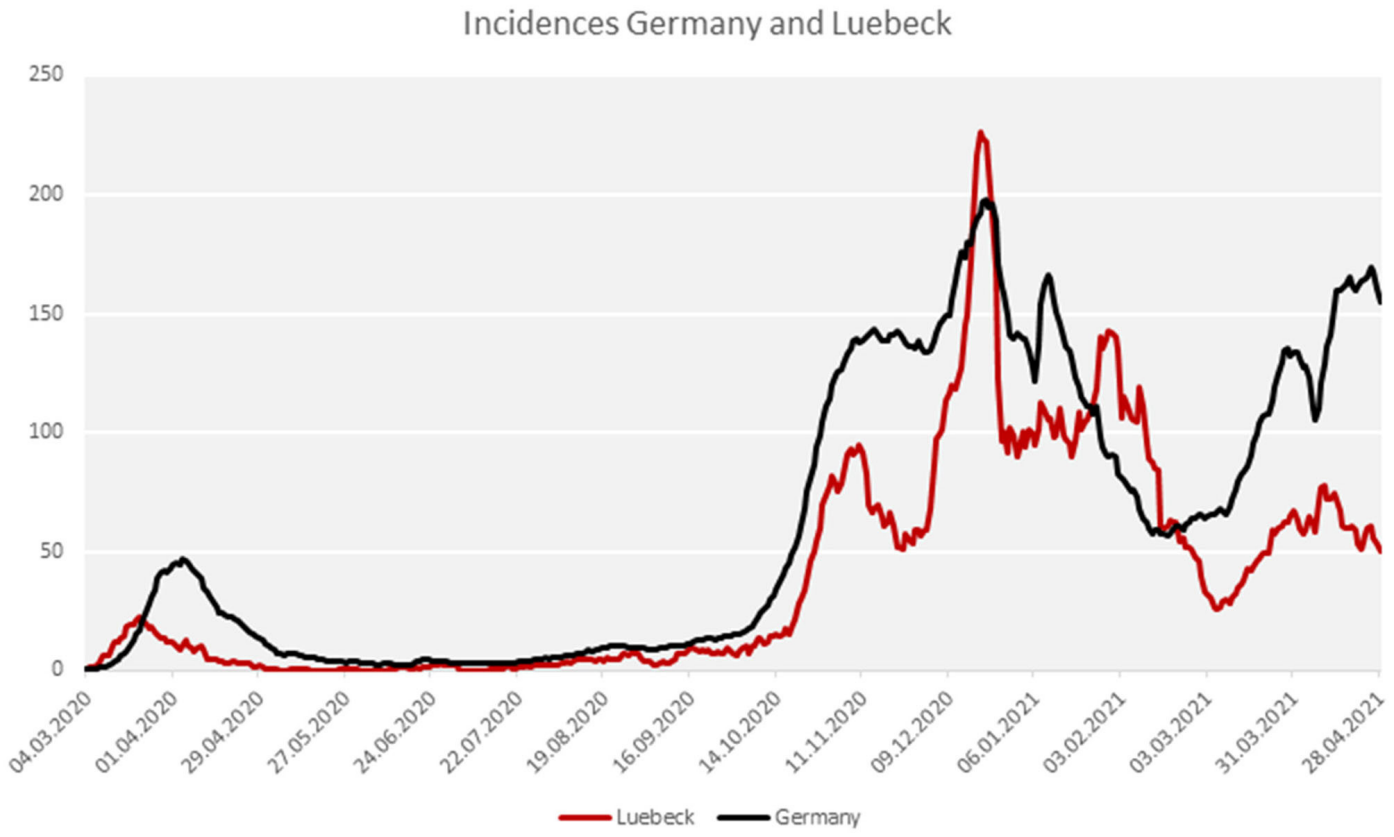
COVID-19 incidence in the city of Luebeck in comparison to Germany.

The study was performed on patients who were notified as index cases to the Health Protection Authority of the City of Luebeck/Germany [approximately 220,000 inhabitants, population density approximately 1,000/m (2)]. With the exception of two major outbreak-related periods in December 2020 and mid-January 2021, the city has been mostly a low-incidence region, when compared with Germany as a whole (Figure 1). The study was performed in compliance with all the relevant ethical regulations and the study was approved by the Institutional Review Board of the University of Luebeck (Germany) (ref. 20-339). Variants of the virus other than wild-type were not detected yet in the study area during the study period.

### Study Population

The data presented here were obtained from the sera of patients that were notified to the local Health Protection Authority as being SARS-CoV-2 positive by PCR irrespective of the clinical manifestation. All of them recovered from the disease without hospitalization. In total, 1,279 patients were invited by e-mail to participate. From the invited patients, 436 responded to the invitation and, finally, 412 of them were eligible for analysis after having given written informed consent and donation of blood (see flowchart). None of the study participants had received a COVID-19 vaccine. 
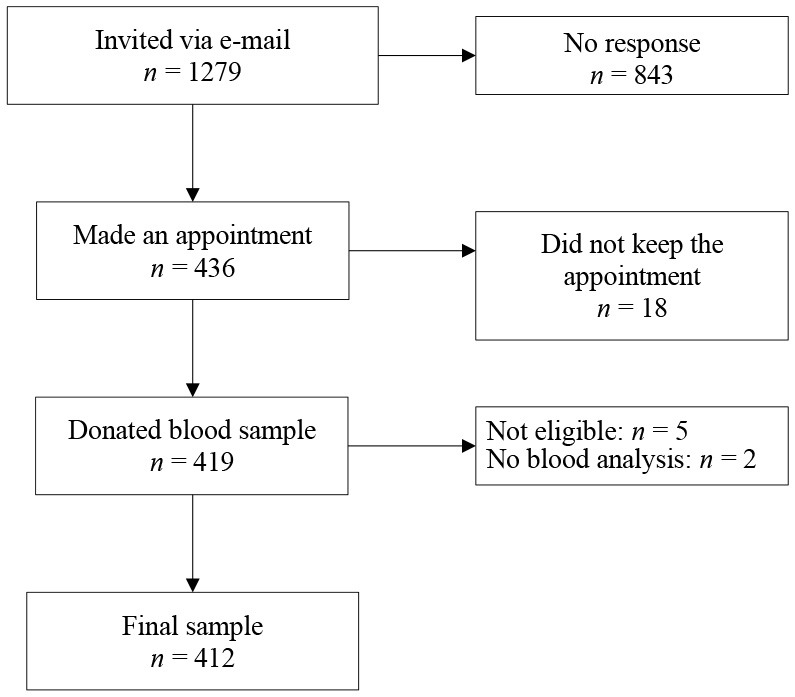


At the study visit, a questionnaire was filled in to assess clinical comorbidities (e.g., obesity, diabetes, autoimmune diseases, hypertonus). COVID-19 disease severity was categorized based on the RKI severity definitions (RKI: klinische Klassifikation der COVID-19 Infektion adaptiert nach WHO Therapeutics and COVID-19: living guideline, https://www.rki.de/DE/Content/Kommissionen/Stakob/Stellungnahmen/Stellungnahme-Covid-19_Therapie_Diagnose.pdf?__blob=publicationFile) and an additional category for patients who did not experience any symptoms of COVID-19 during the infection:

- asymptomatic
- mild (absence of pneumonia)
- moderate (signs of nonsevere pneumonia)
- severe (severe pneumonia, defined as fever and bilateral pulmonary infiltrates and either respiratory rate > 30/min, severe respiratory distress or SpO_2_ < 90–94% on room air)
- critical (acute respiratory distress syndrome; hyperinflammation in conjunction with sepsis or septic shock and multiple organ failure).

### Test Procedures

#### Detection of SARS-CoV-2

Nasopharyngeal swabs were taken from suspected COVID-19 cases by trained personnel either in general practice or in a “drive-in” swab centre run by the Health Protection Authority between March 2020 and December 2020. Swabs were stored in stabilization media and laboratory processed within 4 h. SARS-CoV-2 RNA was detected by using an automated one-step rt-PCR (RIDA^®^GENE SARS-CoV-2 RUO Test; R-Biopharm AG, Darmstadt, Germany; E-gene amplification) run on a RIDA^®^CYCLER according to the instruction of the manufacturer.

### Detection of Anti-SARS-CoV-2 S1-Protein IgG Antibodies

Serum anti-SARS-CoV-2 IgG was detected by automated enzyme-linked immunosorbent assay (product EI 2606-9601 G; EUROIMMUN; https://www.euroimmun.com) according to the instructions of the manufacturer. Signal-to-cut-off (SCO) ratio was calculated as the extinction value (450 nm) of the patient sample divided by the extinction level of the calibrator. A ratio between 0 and < 0.8 was considered as negative, ≥ 0.8 to < 1.1 as borderline, and a ratio ≥ 1.1 as positive. Assay specificity using pre-COVID-19 samples was calculated by the manufacturer as 100%.

### Detection of Neutralizing Antibodies

For detection of neutralizing antibodies, a semiquantitative surrogate virus neutralization test (NeutraLISA from Euroimmun, Product No. 2606-4) was applied, through which the binding of SARS-CoV-2 S1/receptor-binding-domain RBD to ACE2 receptors of the recombinant human host cells is explored. In the first reaction step, samples and controls are incubated with soluble biotinylated ACE. If neutralizing antibodies are present in the sample, they compete with the ACE-receptor for the binding site of the SARS-CoV-2 S1/RBD proteins. Unbound ACE is removed through washing. To detect the bound ACE, a second incubation step with peroxidase-labeled streptavidin is performed, which catalyzes a color reaction. The intensity of the formed color is inversely proportional to the concentration of neutralizing antibodies in the sample. The inhibition (% IH) is calculated by the formula: % IH = 100-(extinction of patient sample × 100/extinction of blank). Values below 20 are considered negative, ≥ 20 to < 35 as borderline, and ≥ 35 as positive. According to the manufacturer, sensitivity and specificity are calculated as 95.9 and 99.7%, respectively.

### Detection of T-Cell Activity

Besides B cells and antibodies, T lymphocytes and cytokines are instrumental for the shaping of the specific acute and memory immune response to SARS-CoV-2 (10). We sought for an easy-to-perform test that might be used as a marker for T-cell responses by determining the capacity to release interferon gamma (IFN-γ) upon specific stimulation. Approximately 7 ml blood was collected in heparinized blood collection tubes. Within 6 h, 0.5 ml of the blood was transferred into three different tubes. One positive control tube (containing a mitogen), one SARS-CoV-2 specific stimulation tube (coated with antigens based on the SARS-CoV-2 spike protein), and one blank tube without antigens to measure individual IFN-γ background of an individual (product ET 2606-3003, https://www.euroimmun.com). After 20–24 h of incubation at 37°C, the tubes were centrifuged at 12.000 rfc for 10 min. IFN-γ concentrations were measured in the supernatants by IFN-γ ELISA according to the instructions of manufacturer (product EQ 6841-9601, EUROIMMUN; https://www.euroimmun.com). When the positive control tube shows a reaction (to confirm sufficient quantity and viability of immune cells), the IFN-γ concentration from the specific stimulation tube (after subtracting the IFN-γ background) was used to quantify specific T-cell responses. Values ≥ 100 mIU/ml were interpreted as borderline, ≥ 200 mIU/ml as positive. It has to be mentioned that all IFN-γ concentrations above the measurement range were replaced by the numerical value of 2,500 mIU/ml. A further quantification was not possible.

### Statistical Analysis

We used standard descriptive statistics to summarize the data. Categorical data were presented as frequencies and percentages and continuous variables were expressed as the mean ± SD. Categorical variables were compared using Fisher's exact test and continuous variables were compared using the Mann-Whitney U test. Pearson's correlation coefficient (Pearson's *R*) and *p*-value were calculated to evaluate the correlation between variables, where a Person's *R* between 0 and 0.19 is regarded as very weak, 0.2 and 0.39 as weak, 0.40 and 0.59 as moderate, 0.6 and 0.79 as strong, and 0.8 and 1 as very strong correlation (11). To identify potential factors associated with SARS-CoV-2 seropositivity (yes/no), a binary logistic regression analysis using the logitem command in Stata (StataCorp, College Station, Texas, USA, version 15) to correct for the specificity and sensitivity of the test was performed. We estimated the models with the following explanatory variables: comorbidity, COVID-19 disease course, and time since the positive COVID-19 test by rt-PCR. Two separate models were run to account for potential confounding. Model 1 included an unadjusted analysis and Model 2 included age- and sex-adjusted analysis. Confounding occurs in epidemiological research when the relationship between a given exposure and a specific outcome (i.e., seropositivity) is distorted (confused) by the influence of a third variable or group of variables (confounders). In this analysis, age and sex were considered confounders if they changed the coefficient of the significant variables by > 10%. The 95% CI for odds ratios (OR) were calculated and a *p* = 0.05 was considered significant. Statistical analyses using were conducted using Stata version 15.0.

## Results

### Sociodemographic Characteristics of the Study Participants

The age of the patients was between 16 and 83 years with a mean age of 44.5 years (SD = ± 16) (Table 1). Of the 412 participants, 235 (57%) were women and 177 (43%) were men (Table 1). Approximately, 40% of the patients reported at least one comorbidity such as obesity, diabetes, autoimmune diseases, or hypertonus (Table 1). Around 90% of the participants reported symptoms during the infection time, 8.9% had no symptoms. About 51% of the patients were classified as having mild disease, 36% as moderate, and only 15 patients (3.6%) had severe disease, but no requirement of hospitalization. Approximately, 9% of all patients completely reported no symptoms. At the study time in January 2021, 41.5% of the patients about 3 months after infection reported still having everyday life-restricting symptoms such as fatigue (21%), disturbance of smell and/or taste (12.5%), and lack of concentration (8%). Nearly, 13% of respondents described more than one persisting symptom (data not shown).

**Table 1:** Sample characteristics (*n* = 412).

**Characteristics**	**N (%)**
**Gender**	
Male	177 (43.0)
Female	235 (57.0)
Mean Age (± SD)	44.5 (16.0)
**Comorbidity**	
Yes	166 (40.3)
No	239 (58.0)
Missing	7 (1.7)
**COVID-19 disease course**	
Asymptomatic	36 (8.7)
Mild disease course	209 (50.7)
Moderate disease course (fever, cough, trouble breathing)	148 (35.9)
Severe disease course	15 (3.6)
Missing	4 (1.0)
**SARS-CoV-2 IgG antibodies**	
Negative	65 (15.8)
Borderline	31 (7.5)
Positive	316 (76.7)
**SARS-CoV-2 neutralizing antibodies**	
Negative	109 (26.5)
Borderline	88 (21.4)
Positive	215 (52.2)
**IGRA**	
Negative	55 (13.3)
Borderline	37 (9.0)
Positive	320 (77.7)
Combined	
Both IGRA and IgG antibodies positive	274 (66.5)
IGRA positive and IgG antibodies negative	46 (11.2)
IgG antibodies positive and IGRA negative	42 (10.2)

### IgG Antibodies Over Time

**Figure 2:**
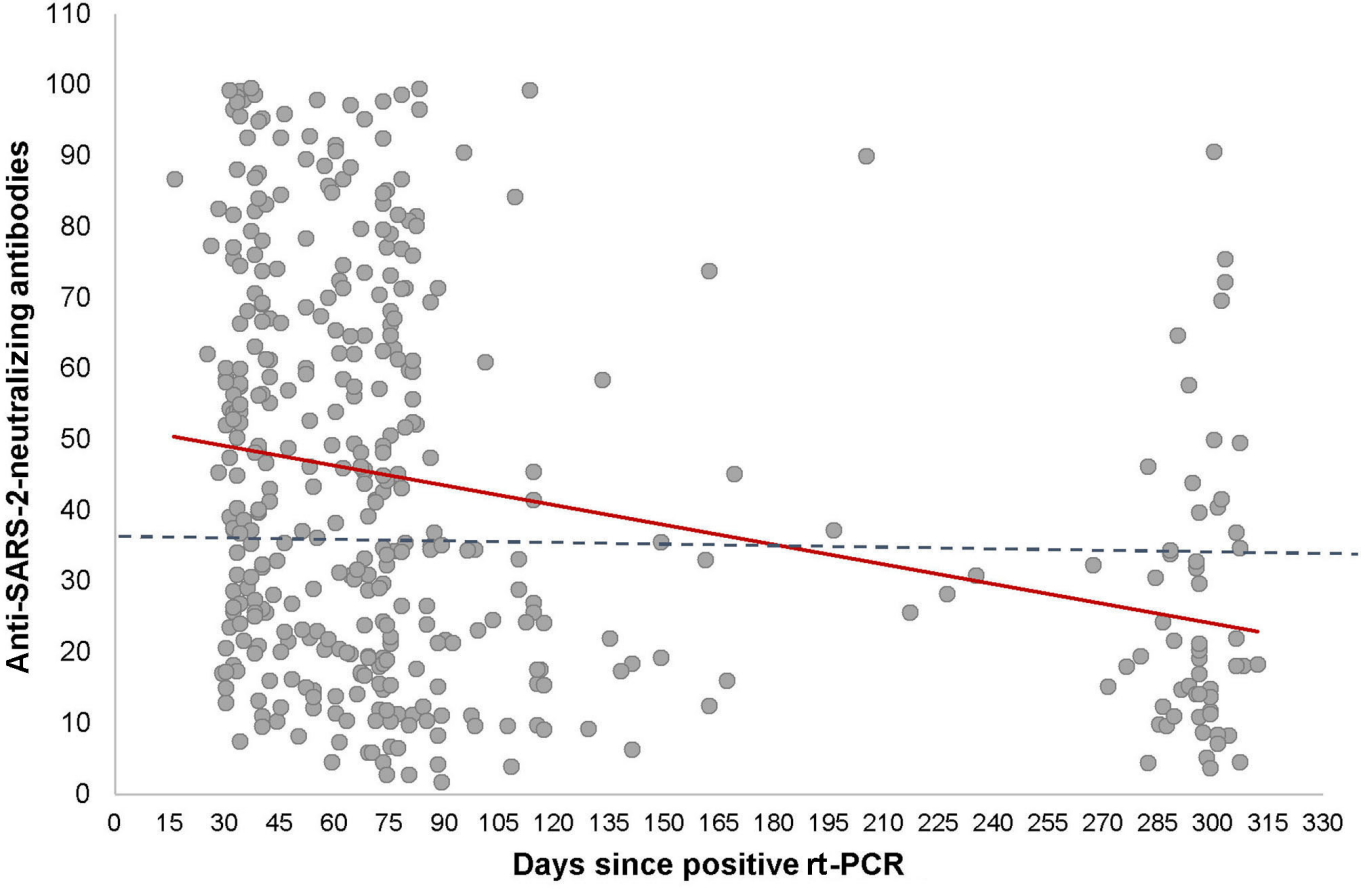
Correlation between anti-SARS-CoV-2-neutralizing antibodies and time after COVID-19 infection. Cross-sectional representation for anti-SARS-CoV-2-neutralizing levels at different time-points for 412 individuals. Each black dot represents one participant and the anti-SARS-CoV-2-neutralizing levels. The red line represents the interpretation line, which shows the negative linear association between the anti-SARS-CoV-2-neutralizing level and the days passed since the diagnosis of COVID-19. The dotted line represents the reference line for the cut-off for having anti-SARS-CoV-2-neutralizing antibodies, which is set at 35.

There was a wide interindividual variation in the antibody levels, supporting the observation from our earlier study. Seropositivity was detected 16 days after the confirmed SARS-CoV-2 diagnosis by PCR (Figure 2). Given that antibody ratios ≥ 1.1 are defined as positive, it is noteworthy that 15.8% (65/412) of the participants did not develop humoral antibodies, despite SARS-CoV-2 detection by PCR. Clinically, the seronegative patients were either asymptomatic, in category 1 (mild) or 2 (moderate). The antibodies had S-protein neutralizing capacity. Most of the sera from the 316 IgG-positive patients (ratio ≥ 1.1) had neutralizing capacity (inhibition index ≥ 20) 215 of 412 (52.2%) participants were neutralizing antibody positive (inhibition index ≥ 20) and 88 (21.4%) participants showed borderline results.

Figure 2 shows the antibody levels for IgG in relation to the days after PCR positivity for the entire sample (*n* = 412). It can be clearly seen that the antibody levels decline over time. There was a moderate but significant negative linear relationship between IgG ratio and the time passed since the positive SARS-CoV-2 diagnosis (*r* = −0.3, *p* < 0.001; Figure 2). Specifically, the mean antibody ratio was the highest in the first 3 months in patients with a severe disease course. In asymptomatic or mild symptomatic patients, the highest mean antibody level, although at a lower level, was observed 1–3 months post COVID-19 infection, with a declining trend in the subsequent time windows (**Table 4**). Thus, the data show that antibody expression is related to the disease severity and that antibody levels fade continuously within approximately 300 days (Figure 2 and **Table 4**).

### Factors Associated With SARS-CoV-2 IgG Seropositivity

To investigate factors associated with SARS-CoV-2 IgG seropositivity, two separate binary logistic regression were conducted (Table 2). Overall, we found that disease severity was positively associated with seropositivity (Model I; OR: 1.69, 95% CI: 1.10–2.84; Table 2), whereas reported having a comorbidities comorbidity had no impact on antibody development (Model I; OR: 1.06, 95% CI: 0.64–1.75). Results also suggest that the likelihood to be seropositive weaned decreases over time. Specifically, the odds of being SARS-CoV-2 antibody positive, 60–119 days after the confirmed COVID-19 diagnosis by PCR, was 0.38 (95% CI: 0.21–0.69). The odds further decreased when 120 or more days had passed since the confirmed COVID-19 diagnosis with an OR of 0.38 in the time period of 60–119 days since PCR positivity. The probability further decreased after 120 days to an OR of 0.19 (Model I; OR: 0.19, 95% CI: 0.09–0.37) (Table 2). Furthermore, when adjusting for age and sex as potential confounders, the variables (i) moderate-to-severe disease course (Model II; OR: 1.77, 95% CI: 1.04–3.01) and (ii) time of diagnosis remained significantly associated with being seropositive (Model II; OR: 0.18, 95% CI: 0.09–0.37) suggesting that. Furthermore, the estimates did not significantly change when adjusting for sex and age. Thus, in the present analysis both age and sex are not confounders that did not significantly confound the association.

**Table 2:** Factors associated with the presence of SARS-CoV-2 IgG antibodies (*n* = 412).

	**Model I** **Crude OR [95% CI]**	**Model II** **Adjusted OR [95% CI]**
**Disease course**		
Asymptomatic/Mild	1.0	1.0
Moderate/Severe	1.69 [1.10 – 2.84]	1.77 [1.04 – 3.01]
**Comorbidity**		
Yes	1.0	1.0
No	1.06 [0.64 – 1.75]	1.07 [0.63 – 1.85]
**Time since positive rt-PCR**		
0–59 days	1.0	1.0
60–119 days	0.38 [0.21–0.69]	0.35 [0.19–0.65]
120 days and more	0.19 [0.09–0.37]	0.18 [0.09–0.37]
**Sex**		
Male	-	1.0
Female	-	1.05 [0.64–1.73]
**Age group**		
16–29 years	-	1.0
30–49 years	-	1.20 [0.63–2.32]
50–69 years	-	0.99 [0.44–1.64]
70 years and older	-	2.91 [0.60–9.65]

*1.0 = reference category; OR, Odd Ratio; CI, Confidential Interval. This table presents the unadjusted (Model I) and the age and sex-adjusted odds ratio (OR) (Model II) of seropositivity (Yes/No) by participant characteristics. The odds ratio was calculated to describe the risk of different groups in positive ELISA serums compared with non-positive ELISA sera. n = 412*.

Although not statistically significant, a tendency for a greater OR of being seropositive was seen among participants having at least one comorbidity (Model II; OR: 1.07, 95% CI: 0.63–1.85) or were of advanced age (Model II; OR: 2.91, 95% CI: 0.60–9.65). For female participants, the OR for being seropositive was almost equal to males (Model II; OR, 1.05, 95% CI: 0.64–1.73) suggesting that being seropositive is equally likely to occur in both female and male participants.

### Factors Associated With SARS-CoV-2 Neutralizing Seropositivity

We also investigated factors associated with SARS-CoV-2 neutralizing seropositivity conducting two separate logistic regression analyses (Table 3). We found that the OR of having antibodies with neutralizing capacity was almost two-fold higher in participants who had a moderate-to-severe COVID-19 disease course as compared to those with asymptomatic or mild disease (Model I; OR. 1.96, 95% CI: 1.18–2.27; Table 3). Similar to IgG SARS-CoV-2 antibodies, neutralizing antibodies elapsed over time and the time passed since the confirmed COVID-19 diagnosis was inversely associated with the probability of expressing neutralizing antibodies, with clear weaning after 60 to 119 days (Model I, OR: 0.39; 95% CI: 0.22–0.70) and even more after 120 days (Model I, OR: 0.15, 95% CI: 0.08–0.31) since the confirmed COVID-19 diagnosis. Individuals for which the time since SARS-CoV-2 testing was more than 120 days ago were less likely to have neutralizing antibodies (OR: 0.14; 95% CI: 0.07–0.28) when compared to those with a confirmed COVID-19 diagnosis made between 0 and 59 days apart from the time of the serological survey. For all variables, the ORs did not change significantly when adjusting for age and sex, suggesting that they do not confound the association between disease course, time since rt-PCR testing and having SARS-CoV-2 neutralizing antibodies. For women, age and gender were not statistically significantly associated with SARS-CoV-2 neutralizing antibody capacity (Table 3). Participants were although not statistically significant. For women, however, the OR for having neutralizing antibodies was slightly decreased below 1, suggesting that they might be less likely to develop SARS-CoV-2 neutralizing antibodies (Model II; OR, 0:94; 95% CI: 0.58–1.52).

**Table 3:** Factors associated with the presence of SARS-CoV-2 neutralizing antibodies (*n* = 412).

	**Model I** **Crude OR [95% CI]**	**Model II** **Adjusted OR [95% CI]**
**Disease course**		
Asymptomatic/Mild	1.0	1.0
Moderate/Severe	1.96 [1.18 – 2.27]	2.07 [1.23–3.47]
**Comorbidity**		
Yes	1.0	1.0
No	1.39 [0.85 – 2.28]	1.34 [0.79–2.27]
**Time since positive RT-PCR**		
0–59 days		1.0
60–119 days	0.39 [0.22–0.70]	0.35 [0.14–0.63]
120 days and more	0.15 [0.08–0.31]	0.14 [0.07–028]
**Sex**		
Male		1.0
Female		0.94 [0.58–1.52]
**Age group**		
16–29 years		1.0
30–49 years		1.02 [0.55–1.89]
50–69 years		0.93 [0.53–1.78]
70 years and older		2.56 [0.84–10.11]

*1.0 = reference category; OR, Odd Ratio; CI, Confidential Interval. This table presents the unadjusted (Model I) and the age and sex-adjusted odds ratio (OR) (Model II) of having neutralizing antibodies (Yes/No) by participant characteristics. The odds ratio was calculated to describe the factors of different groups in positive neutralizing antibody assays compared with negative neutralizing antibody assays*.

### T-Cell Activity Over Time

Since T cells are instrumental for the development of the S-protein reactive B-cell activity, we explored T-lymphocyte activity. We chose to investigate the induction and release of IFN-γ upon S-protein specific stimulation. This assay reflects an easy-to-perform summarized image of T-lymphocytes activity without considering all subgroups of T-lymphocytes or other IFN-γ-producing cells. As can be seen in Table 1, 320 of 412 (77.7%) of the PCR-positive patients had a positive Interferon-gamma-release assay (IGRA) test result (≥ 200 mIU/ml), while 37 of 412 (9%) were borderline. A strong correlation between the IFN-γ levels and the time passed since the positive SARS-CoV-2 diagnosis by PCR (*r* = 0.6, *p* < 0.001).

**Figure 3:**
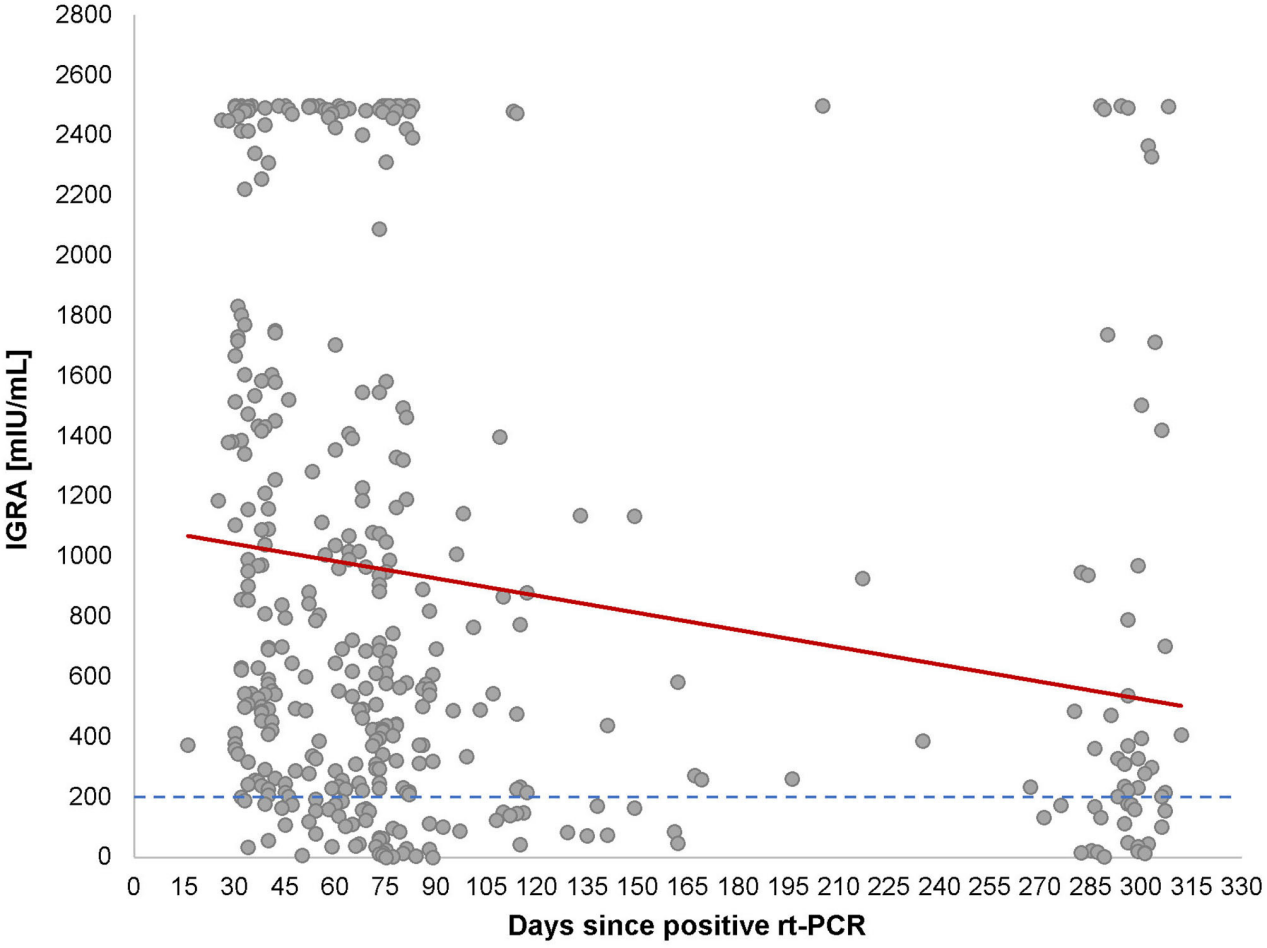
Correlation between Interferon Gamma Release Assay (IGRA) and time after COVID-19 infection. Cross-sectional representation for IGRA values at different time-points for 320 individuals. Each black dot represents one participant and the IGRA milli-international units per milliliter (mIU/ml) and the red line represents the interpretation line, which shows the negative linear association between IGRA values and the days passed since the diagnosis of COVID-19. The dotted line represents the cut-off of IGRA values at 200 (mIU/ml).

**Figure 4:**
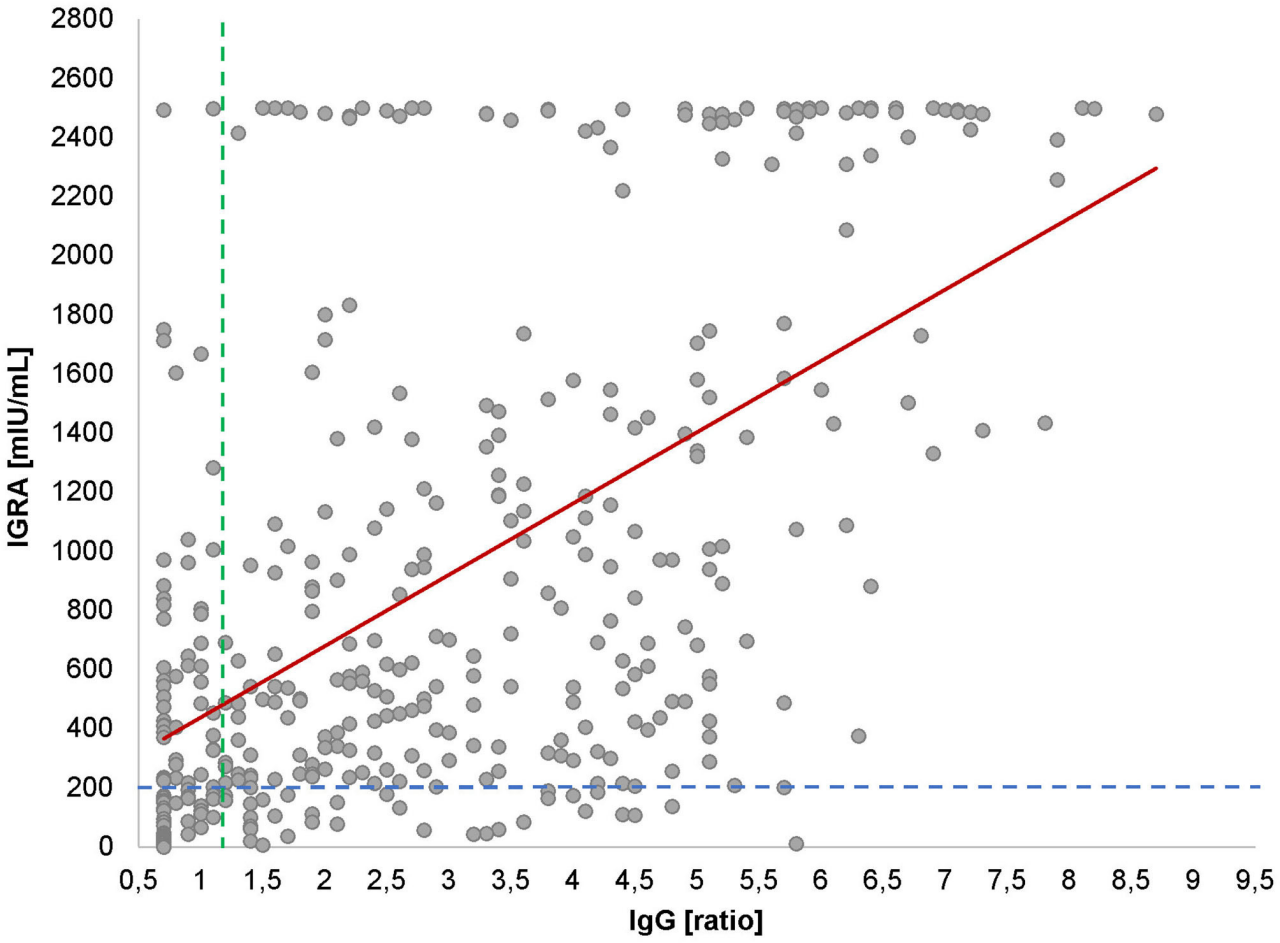
IgG antibodies vs. IGRA. Cross-sectional scatter plot for IGRA and IgG values. Each black dot represents one participant and the respective IgG and IGRA values. The dotted line represents the cut-off for positive IgG values at 1.1 (vertical line) and for IGRA values at 200 mIU/ml (horizontal line). The red line shows the significant positive correlation between IgG and IGRA values (*r* = 0.6, *p* < 0.001).

Looking at the correlation between antibody levels and IFN-γ concentrations, a heterogeneous picture emerged (Figure 4). While in most cases both values were concordant, there were a considerable number of cases with high to very high IFN-γ levels and low-antibody levels and vice versa. Looking at the cloud of dots, it is striking that there appears to be a population of patients whose cells produce extremely high levels of IFN-γ (>2,500 mIU/ml), regardless of the antibody response (Figure 3) or the post-SARS-CoV-2 diagnosis (Figure 2).

In summary, in our SARS-CoV-2 PCR-positive cohort, the antibody profile was heterogeneous. In most cases, antibodies could be detected between 16 and 73 days after the determination of a SARS-CoV-2 infection *via* PCR from a nasopharyngeal swab. The antibody levels continuously decreased over time.

**Figure 5:**
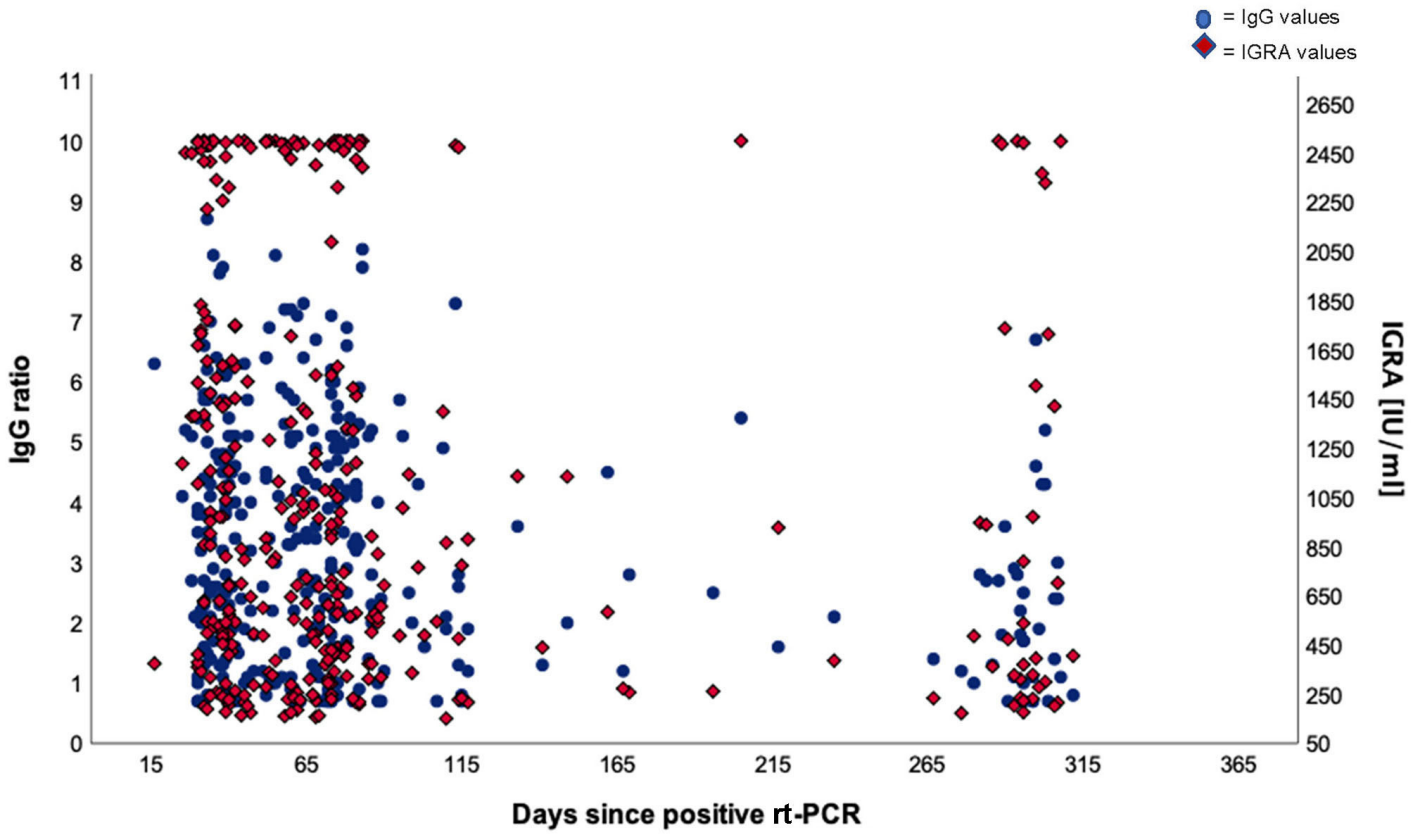
Dual axis scatter plot for IgG ratios and IGRA values. The dots represent participants and their positive IgG values (cut-off at 1.1) and the diamonds represent participants and their positive IGRA values (cut-off at 200 mIU/ml).

In 65 of 412 patients (15.8%), no significant antibody levels could be detected in two up to four consecutive analyses between days 16 and 73. As far as the IFN-γ release is concerned, a similar picture emerged. The levels were heterogeneous and decreased over time. Noteworthy, a significant part of the patients produced very high levels of IFN-γ irrespective of the concentration of antibodies measured (Figure 5).

## Discussion

In this study, we evaluated the development of specific humoral and cellular immune responses in outpatients recovered from SARS-CoV-2 infection, mostly with mild-to-moderate disease (Table 1) in a cross-sectional study design. Therefore, we chose to determine serum IgG antibodies and IFN-γ release in response to the viral spike (S) glycoprotein in view of the time that had passed since the infection. At the time of the study only wild-type virus was detected in our study area. Variants such as beta or delta had not been detected in our area so far.

In this study, about 16% of the patients had neither positive nor borderline detectable IgG antibodies at the last study visit. These data substantiate our previous findings from the first wave of the pandemic in early 2020 (9). On a population basis, the clinical severity of the disease was positively correlated with the level of SARS-CoV-2 neutralizing antibodies (Tables 3, 4), as had been shown previously by others (12). On an individual level, there was great variability between patients. Thus, the individual level of antibodies is not of diagnostic value, for example, for the assessment of patients with long COVID syndrome, which is often associated with chronic fatigue (13). As expected, in many cases, the antibody levels faded over time. When compared with the first 3 months after infection, the mean antibody levels decayed steadily and approximately halved within 300 days (Figure 2). Although our data do not allow a meaningful calculation of the half-life due to a cross-sectional design instead of longitudinal for each patient, the finding is in accordance with reports from others (14–17), who calculated the half-life of neutralizing IgG antibodies with 140 to 220 days. Recently, colleagues reported that IgG antibodies to nucleocapsid protein reduced the risk of reinfection for up to 10 months after primary infection (18). Researchers from the University of Padua and Imperial College London tested more than 85% of the 3,000 residents of Vo', Italy, in February/March 2020 for infection with SARS-CoV-2 and tested them again in May and November 2020. The strength of the immune response did not depend on the symptoms and the severity of the infection. The team found that 98.8% of people infected in February/March showed detectable levels of antibodies in November, and there was no difference between people who had suffered symptoms of COVID-19 and those who had been symptom-free. The results showed that while all antibody types showed some decline between May and November, the rate of decay was different depending on the assay (19). Interestingly, after COVID vaccination, neutralization capacity was different from natural infection (20).

**Table 4:** Mean antibody ratio since confirmed COVID-19 diagnosis and disease severity.

	**Days since confirmed COVID-19 diagnosis**	
**Disease course**	**1–3 months**	**3 and more months**
	**Mean (± SD)**	**Mean (± SD)**
Asymptomatic/Mild	3.38 (2.56)	1.78 (1.53)
Moderate/Severe	4.28 (2.22)	2.26 (1.87)

At the time the study was done, no variants were detected in the area of Luebeck. We are therefore sure that our neutralization assays did not fail due to undetected virus variants.

Very few published data sets compare antigen-specific B-cell and T-cell immunity. We, therefore, examined interrelationships between IgG antibody levels and IFN-γ release. Like with the antibody kinetics, the IFN-γ values decayed over time after infection with similar kinetics (Figure 3) which is in line with other works (21). Unexpectedly, however, we saw a substantial number of patients with low-antibody levels and extremely high-IFN-γ levels and vice versa (Figure 4). Although unlikely, it cannot be completely ruled out that the observed T-cell reactivity was due to pre-existing memory of T cells recognizing the common cold coronaviruses, as has been described before (22, 23). Existing T cells might be an explanation for cross-reactions in an IGRA that could also be seen in our study (24). To explain this dichotomy, an in-depth longitudinal analysis of the precise numbers and types of IFN-γ producing cells will be necessary, especially the characterization of T memory cells.

Since our data and those from others show that determination of antibodies alone is not predictive for protection against SARS-CoV-2 disease, simultaneous determination of IFN-γ may be a valuable adjunct and may also predict the time-point for possible necessary booster vaccinations on an individual basis. The antigens used are based on the S1 of the wild type—this is the antigenic origin currently used by all well-known manufacturers. We mainly conclude from our data that using antibody, neutralizing and IFN-γ assays are useful to monitor individual immune responses. However, we know that in infections with the beta or delta variant, for example, there are mutations that lead to a kind of immune escape. Theoretically, it cannot be ruled out that in infections with highly divergent variants, differences in immune responses against the S1 used would be observed.

### Strengths and Limitations

This study has few limitations that must be acknowledged. Longitudinal data for each subject, with at least three time-points per subject, would be required to better understand the kinetics of durability of SARS-CoV-2-specific antibodies. Furthermore, since patients were sampled only once at various time-points after the confirmed COVID-19 diagnosis, the results are vulnerable to bias caused by individual variations and inter-individual variation cannot be excluded. Nevertheless, the current cross-sectional data describe well the dynamics of spike-specific antibodies over 10 months and IFN-γ release by blood T lymphocytes at one study point. This study was not sufficiently powered to control for many variables simultaneously.

## Conclusion

This study shows that on average 9.8 months after detecting SARS-CoV-2 in nasopharyngeal specimen, we found a significant weaning of IgG antibodies levels against the viral S-protein in comparison to the initial values. Most of the patients showed robust IFN-γ production after S-protein stimulation of peripheral blood cells, indicating the importance of T lymphocytes for shaping the protective immune reaction. However, in a substantial proportion of our samples, we found low-antibody levels accompanied with high-IFN-γ levels and vice versa. For future assessment of protection and possible vaccination strategies, both, determination of antibodies and IFN-γ is recommended.

## Data Availability Statement

The original contributions presented in the study are included in the article, further inquiries can be directed to the corresponding author.

## Ethics Statement

The ethical committee of the University of Luebeck approved the study (ref. 20-339). The patients/participants provided their written informed consent to participate in this study.

## Author Contributions

JS, IB, JR, AM, and WS wrote the article. JS, IB, DZ, JK, and WS were responsible for data analysis. SS, TM, and JR were responsible for the recruitment of patients, data collection, and contributed to data analysis. AM and WS designed the study. All authors approved the final version of the manuscript.

## Funding

This study was funded by the City of Luebeck and EUROIMMUN AG.

## Conflict of Interest

The authors declare that the research was conducted in the absence of any commercial or financial relationships that could be construed as a potential conflict of interest.

## Publisher's Note

All claims expressed in this article are solely those of the authors and do not necessarily represent those of their affiliated organizations, or those of the publisher, the editors and the reviewers. Any product that may be evaluated in this article, or claim that may be made by its manufacturer, is not guaranteed or endorsed by the publisher.

## Abbreviations

COVID-19: coronavirus disease 19
CI: confidence interval
ELISA: Enzyme-linked Immunosorbent Assay
IgG: immunoglobulin G
IGRA: Interferon-Gamma-Release Assay
rt-PCR: real-time polymerase chain reaction
SARS-CoV-2: Severe acute respiratory syndrome coronavirus 2.
